# Role and mechanism of ribosomal protein S3 in tyrosine kinase inhibitor resistance in chronic myeloid leukemia

**DOI:** 10.3389/fonc.2026.2005945

**Published:** 2026-10-29

**Authors:** Zheng Sun, Sijin Liu, Yuxin Ning, Jinwen Dang, Zhuanghui Hao, Xiaoning Wang, Bianbian Qiao, Qianni Jin, Kai Dong, Congrui Yan, Zhilei Jin, Hongwei Wang, Zhifang Xu

**Affiliations:** Key Laboratory of Molecular Diagnosis and Treatment of Hematological Diseases of Shanxi Province, Department of Hematology, The Second Hospital of Shanxi Medical University, Taiyuan, Shanxi, China

**Keywords:** chronic myeloid leukemia, drug resistance, NF-κB, RPS3, tyrosine kinase inhibitors

## Abstract

**Introduction:**

Resistance to tyrosine kinase inhibitors (TKIs) remains a major cause of treatment failure in chronic myeloid leukemia (CML). This study investigated the role of ribosomal protein S3 (RPS3) in TKI resistance and its association with NF-κB signaling.

**Methods:**

Exploratory RNA sequencing and an RNA-binding protein database were used to prioritize a candidate for subsequent clinical expression and functional analyses. RPS3 expression was examined in an independent clinical cohort and resistant cell models. RPS3 knockdown and pharmacological modulation were used to evaluate drug sensitivity, apoptosis, cell cycle distribution, and NF-κB-associated signaling.

**Results:**

RPS3 was upregulated in clinical samples from patients with TKI resistance or poor responses and in resistant cell models. Receiver operating characteristic analysis showed that RPS3 expression discriminated between resistant/poor-response patients and good responders in the validation cohort. RPS3 knockdown lowered imatinib and nilotinib IC_50_ values, with increased apoptosis and G_0_/G_1_-phase arrest following TKI exposure. These effects were accompanied by reduced p65 phosphorylation, a lower p65 nuclear/cytoplasmic fluorescence ratio, and decreased MCL-1 and BCL-XL mRNA levels. TNF-α treatment increased TKI tolerance and reduced TKI-induced apoptosis in RPS3-knockdown cells, whereas co-treatment with BAY-11-7082 counteracted these effects. BAY-11-7082 also showed synergistic inhibitory effects with TKIs in resistant cells under the highest single agent model.

**Discussion:**

These findings identify RPS3 as a contributor to TKI resistance in CML cell models and connect its functional effects to NF-κB-associated anti-apoptotic signaling. By integrating clinical expression data with functional and pharmacological analyses, this study supports further investigation of RPS3 as a candidate target for sensitizing resistant CML cells to TKIs.

## 1 Introduction

Chronic myeloid leukemia (CML) is a myeloproliferative neoplasm caused by malignant clonal expansion of hematopoietic stem/progenitor cells. Its molecular hallmark is the BCR::ABL1 fusion gene generated by the t(9;22)(q34;q11.2) chromosomal translocation (1–3). The introduction of tyrosine kinase inhibitors (TKIs) has markedly improved the long-term prognosis of patients with CML and has made sustained disease control possible for many patients (4–7). Nevertheless, a subset of patients still develop suboptimal molecular responses, acquired resistance, or disease progression during treatment. TKI resistance remains an important clinical problem that limits the therapeutic efficacy of CML treatment (6, 8–12).

The mechanisms of TKI resistance in CML can be broadly classified into BCR::ABL1-dependent and BCR::ABL1-independent mechanisms. BCR::ABL1 kinase domain mutations, BCR::ABL1 amplification, and altered drug binding are classical mechanisms of resistance (8, 9). However, in a substantial proportion of resistant patients, BCR::ABL1 mutations alone cannot fully explain treatment failure (10, 11). Recent studies have shown that BCR::ABL1-independent mechanisms, including persistent survival of leukemia stem cells, protection by the bone marrow microenvironment, enhanced drug efflux, epigenetic remodeling, and abnormal activation of bypass pro-survival signaling pathways, also contribute to the development of CML resistance (10–14). Therefore, identifying novel BCR::ABL1-independent regulators of resistance is important for elucidating the biology of CML drug resistance, discovering potential therapeutic targets, and optimizing combination treatment strategies.

RNA-binding proteins (RBPs) regulate RNA splicing, transport, stability, localization, and translation and thereby influence gene expression and cell fate (15, 16). Increasing evidence indicates that aberrant expression of RBPs is closely associated with tumor cell proliferation, stemness maintenance, differentiation arrest, and therapeutic resistance (17). For example, circSETD3 has been reported to promote acquired gefitinib resistance in non-small cell lung cancer through an FXR1/ECT2 pathway, illustrating an RNA-regulatory mechanism involving an RBP (18). In hematologic malignancies, Musashi-2 (MSI2) promotes the progression of myeloid leukemia and maintains leukemia stem cell properties, and its high expression is associated with disease progression and poor prognosis (19, 20). Splicing-related RBPs such as RBM39 have also been shown to be critical dependencies for leukemia cell survival and have emerged as potential therapeutic targets (21). However, the role of RBPs in CML TKI resistance remains insufficiently characterized, particularly whether RBPs participate in resistance by regulating BCR::ABL1-independent pro-survival pathways.

Ribosomal protein S3 (RPS3) is an important component of the eukaryotic 40S ribosomal subunit. In addition to its canonical ribosomal function, RPS3 has multiple extra-ribosomal functions, including roles in DNA damage repair, oxidative stress responses, apoptosis regulation, and modulation of transcription factor activity (22, 23). Previous studies have shown that RPS3 can function as a non-Rel subunit of the NF-κB transcriptional complex, interacting with p65 and enhancing the transcriptional activity of NF-κB target genes (24). NF-κB signaling is a key pro-survival pathway in tumor cells. Its sustained activation can upregulate multiple anti-apoptotic molecules and increase resistance to drug-induced apoptosis (25–27). In CML, aberrant NF-κB activation has also been implicated in leukemia cell survival and TKI resistance (28–30), suggesting that the RPS3/NF-κB axis may contribute to the regulation of CML drug resistance.

RPS3 has been implicated in cell growth, evasion of apoptosis, and therapeutic resistance in several cancers. Reported examples include metastasis and cisplatin resistance associated with STAT1/NF-κB signaling in adenoid cystic carcinoma (31), regulation of XIAP expression in breast cancer (32), and proteasome inhibitor resistance in multiple myeloma (33). In the latter study, Chen et al. linked TRIP13/PKCδ-mediated RPS3 phosphorylation to canonical NF-κB activation, providing a hematologic precedent for examining RPS3-associated drug resistance. These findings raise the question of whether RPS3 also contributes to acquired TKI resistance in CML and whether this contribution involves NF-κB-associated survival signaling. We addressed this question by integrating exploratory RNA sequencing and RBP screening with an independent clinical expression cohort, established resistant cell models, and functional perturbation of RPS3. We then examined p65 phosphorylation and nuclear/cytoplasmic distribution, anti-apoptotic gene expression, and responses to pharmacological modulation of NF-κB signaling. This design links the clinical association of RPS3 expression with treatment response to its functional contribution to TKI sensitivity in resistant CML cells.

## 2 Materials and methods

### 2.1 Cell culture

The human CML cell line K562 was obtained from the Cell Bank of the Chinese Academy of Sciences, Shanghai, China. In our previous work, drug-resistant cell lines were established by long-term drug pressure selection with gradual adjustment of drug concentrations. K562-IMR cells that could stably grow in 1 μmol/L imatinib, K562-NIR cells that could stably grow in 50 nmol/L nilotinib, and K562-(IM+NI)R cells that could stably grow in 1 μmol/L imatinib plus 100 nmol/L nilotinib were ultimately obtained. These cells were maintained in RPMI-1640 medium (Bio-channel, China) supplemented with 10% fetal bovine serum (Bio-channel, China). HEK293T cells were cultured in Dulbecco’s modified Eagle’s medium (DMEM; Bio-channel, Nanjing, China). All cells were incubated at 37 °C in a humidified atmosphere containing 5% CO_2_.

### 2.2 Transcriptome sequencing analysis

Exploratory transcriptome sequencing included 11 patients with chronic-phase CML: 3 newly diagnosed patients (DIA), 3 good responders after TKI treatment (GR), and 5 patients with resistance or poor molecular responses (R). All patients in the sequencing and clinical validation cohorts were in chronic phase. Molecular responses were interpreted with reference to the ELN 2020 recommendations (6). GR patients had favorable responses during TKI treatment, BCR::ABL1^IS^ ≤0.1% at sampling, and no documented loss of major molecular response (MMR) in the available records. The R group included patients with an inadequate molecular response or a history of MMR loss during TKI treatment. For the R-group patients with treatment changes, clinical record review confirmed that response-related switches followed loss of MMR; the documented intolerance-related switch was recorded separately in **Supplementary File** CML_patient—Additional clinical information. Intolerance alone was not used as evidence of resistance. Historical response information was retained for classification even when BCR::ABL1^IS^ was ≤0.1% at sampling. Available clinical characteristics and TKI histories are summarized in **Supplementary File** CML_patient—Additional clinical information. Bone marrow or peripheral blood mononuclear cells (MNCs) were isolated by density-gradient centrifugation. Total RNA was extracted and sequenced on an Illumina platform, with filtering and quality control of the raw data.

### 2.3 Differential gene screening

Differential expression analysis was performed using DESeq. Subsequent resistance-focused analyses used the R-versus-GR comparison (five versus three patients). Genes with nominal *P*-values <0.05 were intersected with the RNA-Binding Protein DataBase (RBPDB; https://rbpdb.ccbr.utoronto.ca/index.php) using Venny 2.1.0, yielding 28 exploratory RBP-associated candidates. Follow-up focused on the five candidates with higher expression in R than in GR. RPS3 was prioritized based on its high normalized read-count abundance and published evidence linking it to survival signaling and drug resistance. Expression summaries and nominal and adjusted *P*-values are provided in **Supplementary Table S1**.

### 2.4 Real-time quantitative PCR

Real-time quantitative PCR (RT-qPCR) was used to measure target-gene expression, with GAPDH as the internal control. Primer sequences are provided in **Supplementary Table S2**. Total RNA was extracted using TRIzol reagent (Takara, Japan) and reverse-transcribed into cDNA using 5× PrimeScript RT Master Mix (Takara, Japan). RT-qPCR was then performed using TB Green Premix Ex Taq II (Tli RNaseH Plus; Takara, Japan). GAPDH was used as the internal control, and relative gene expression was calculated using the 2^−ΔΔCt^ method. RPS3 mRNA expression was further validated in bone marrow or peripheral blood MNC samples from an independent clinical cohort consisting of 30 patients in the resistant/poor-response group and 32 patients in the good-response group.

### 2.5 Cell transfection

shRNAs targeting RPS3 and the corresponding negative control shRNA (sh-NC) were synthesized by PPL (China). Lipofectamine 293 (Invitrogen, USA) was used for transfection of shRNA and plasmid vectors. Transfection efficiency was confirmed by RT-qPCR and western blotting. The shRNA sequences are listed in **Supplementary Table S2**.

### 2.6 Cell proliferation assay

After infection, logarithmically growing empty-vector control cells and knockdown cells were collected and adjusted to the appropriate density. Cells were seeded into 96-well plates at 1 × 10^4^ cells/well in 100 μL medium, with blank wells included. Four technical replicate wells were included per group. Based on preliminary experiments, resistant cell lines were treated with different types and concentration gradients of TKIs. After incubation at 37 °C for 48 h, 10 μL of CCK-8 reagent (NCM Biotech, China) was added to each well, and the plates were incubated in the dark for an additional 2.5 h. Absorbance at 450 nm was measured using a microplate reader to assess relative cell viability.

### 2.7 Flow cytometric analysis of apoptosis

Cells from each treatment group were collected and stained using an Annexin V/7-AAD apoptosis detection kit (Multi Sciences, China) according to the manufacturer’s instructions. Briefly, cells were washed with PBS and resuspended in an appropriate volume of 1× Binding Buffer. Annexin V conjugate and 7-AAD staining solution were added, and the cells were incubated at room temperature in the dark before analysis using a flow cytometer (Beckman Coulter NAVIOS, USA). Annexin V-positive/7-AAD-negative cells were defined as early apoptotic cells, whereas Annexin V-positive/7-AAD-positive cells were defined as late apoptotic/dead cells. The total apoptosis rate was calculated as the sum of early and late apoptotic/dead cells.

### 2.8 Flow cytometric analysis of cell cycle distribution

Cell cycle distribution was analyzed using a Cell Cycle kit (Multi Sciences, China) according to the manufacturer’s instructions. A total of 2 × 10^5^ to 1 × 10^6^ cells were collected, washed with PBS, and incubated with DNA Staining Solution and Permeabilization Solution. After vortexing, the cells were incubated at room temperature in the dark for 30 min. DNA content was then analyzed using a flow cytometer (Beckman Coulter NAVIOS, USA), and the proportions of cells in the G_0_/G_1_, S, and G_2_/M phases were quantified. Statistical analyses were performed using GraphPad Prism software.

### 2.9 Western blotting

A total of 1 × 10^6^ to 2 × 10^6^ cells were collected and washed three times with pre-cooled PBS. Cells were lysed in RIPA lysis buffer (150 mM NaCl, 1.0% NP-40, 0.5% sodium deoxycholate, 0.1% sodium dodecyl sulfate, and 50 mM Tris, pH 8.0) supplemented with protease inhibitors and 1 mM PMSF (Boster, China) for 30 min. Lysates were centrifuged at 12,000 rpm for 15 min at 4 °C, and the supernatants were collected. Protein concentrations were determined using a BCA protein assay kit (Solarbio, China). Equal amounts of protein were separated on 10% SDS-PAGE gels and transferred onto pure nitrocellulose membranes (Millipore, USA). Membranes were blocked with 5% non-fat milk at room temperature for 2 h and incubated with primary antibodies overnight. The next day, membranes were incubated with HRP-conjugated secondary antibodies for 1.5 h. Finally, ultrasensitive ECL chemiluminescent reagent (Seven, China) was added, and protein bands were detected using a chemiluminescence gel imaging system (Bio-Rad ChemiDoc MP, USA). Antibody and reagent details are provided in **Supplementary Table S3**.

### 2.10 Immunofluorescence

Logarithmically growing cells from each group were washed with PBS, fixed with 4% paraformaldehyde, permeabilized, and blocked with BSA. Cells were incubated with an anti-p65 primary antibody overnight at 4 °C. After washing with PBS, fluorescently labeled secondary antibodies were added and incubated at room temperature in the dark. Nuclei were then stained with DAPI, and the cells were mounted with anti-fade mounting medium. Images were acquired using a digital scanner or fluorescence microscope and analyzed using ImageJ. A uniform fluorescence threshold was applied across the images being compared. For each cell, integrated p65 fluorescence intensity (IntDen) was measured in the nuclear and whole-cell regions. Cytoplasmic IntDen was calculated by subtracting nuclear IntDen from whole-cell IntDen. The p65 nuclear/cytoplasmic ratio was calculated as nuclear IntDen/(whole-cell IntDen − nuclear IntDen). This dimensionless ratio represents the relative distribution of integrated p65 fluorescence between the nucleus and cytoplasm. Fifty cells per group were quantified in each independent experiment, and their individual ratios were averaged to obtain one value per group for that experiment. Three independent experiments were performed, with cell culture, treatment, staining, and imaging conducted separately for each experiment. Each experiment included the corresponding shNC and shRPS3 groups. Thus, *n* = 3 refers to independent biological experiments rather than replicate images or individual cells.

### 2.11 NF-κB activation and inhibition

TNF-α (Yeasen Biotechnology, China) and BAY-11-7082 (MCE, USA) were used as an NF-κB pathway activator and inhibitor, respectively. Logarithmically growing cells were adjusted to the appropriate density and seeded into 96-well plates or plates for flow cytometric assays. According to the experimental grouping, cells were treated simultaneously with the corresponding TKIs, TNF-α, and BAY-11–7082 at the indicated final concentrations. K562-IMR-shRPS3 cells were treated with imatinib, whereas K562-NIR-shRPS3 cells were treated with nilotinib. The final concentrations of TNF-α were 10 ng/mL and 20 ng/mL, and the final concentration of BAY-11–7082 was 1.5 μM. After incubation at 37 °C in 5% CO_2_ for 48 h, cell viability was measured by the CCK-8 assay and IC_50_ values were calculated. Apoptosis was detected by Annexin V/7-AAD double staining and flow cytometry. All experiments were performed in at least three independent replicates.

TNF-α and BAY-11–7082 were added simultaneously with the TKIs; no TNF-α prestimulation was performed.

### 2.12 Drug combination and synergy analysis

To evaluate the effects of combined treatment with the NF-κB inhibitor BAY-11–7082 and TKIs on TKI-resistant CML cells, K562-IMR and K562-NIR cells were seeded into 96-well plates and treated with different concentration gradients of imatinib or nilotinib in combination with BAY-11-7082. After 48 h of drug treatment, cell viability was measured using the CCK-8 assay, and cell survival was calculated relative to the untreated control group.

Drug synergy was analyzed using SynergyFinder software (34, 35). Cell viability data corresponding to each drug concentration combination were imported into SynergyFinder, and the highest single agent (HSA) model was selected to calculate synergy scores and generate synergy heat maps. According to commonly used SynergyFinder interpretation criteria, a synergy score >10 indicates synergy, a score between −10 and 10 indicates an additive effect, and a score <−10 indicates antagonism.

### 2.13 Statistical analysis

All experimental data were statistically analyzed and plotted using GraphPad Prism 10.1. Quantitative data are presented as the mean ± standard deviation (mean ± SD). All cell-based experiments were performed in at least three independent replicates. For p65 nuclear/cytoplasmic ratio analysis, the mean of the 50 single-cell ratios obtained for each group in each independent experiment was used as the statistical unit; individual cells were not treated as independent biological replicates. For these imaging analyses, three independent biological experiments were performed as described above.

Comparisons between two independent groups were performed using an unpaired, two-tailed Student’s *t*-test. Comparisons of three or more groups with a designated control were performed using ordinary one-way analysis of variance (ANOVA), followed by Dunnett’s multiple-comparisons test. Adjusted *P*-values were used for these control-versus-treatment comparisons. The corresponding control was parental K562 for comparisons among parental and resistant cell models, shNC for comparisons of knockdown groups with their control, or the shRPS3+TKI group without TNF-α or BAY-11–7082 for the pharmacological experiments. Cell cycle phase distributions were analyzed separately for each cell model using ordinary two-way ANOVA with treatment group and cell cycle phase as the factors, including their interaction, followed by Tukey’s multiple-comparisons test comparing the four treatment groups within each phase. Each phase constituted a separate family of six pairwise comparisons.

For drug sensitivity assays, IC_50_ values were estimated by nonlinear regression using the “[Inhibitor] vs. normalized response—Variable slope” model in GraphPad Prism, with concentration entered on its original scale, the upper and lower plateaus fixed at 100 and 0, respectively, and a fitted Hill slope. Group comparisons were performed on the resulting IC_50_ values using the two-group or multigroup tests described above. ROC curves were used to evaluate the ability of RPS3 expression to distinguish patients in the study-defined resistant/poor-response group from good responders, and the area under the curve (AUC), 95% confidence interval (95% CI), and *P*-value were calculated. Drug combination synergy was analyzed using SynergyFinder software based on the HSA model (34, 35). IC_50_ values are summarized in **Supplementary Table S4**.

For the supplementary demographic comparisons, age was analyzed using a two-sided Mann–Whitney *U* test with tie correction and continuity correction and sex using a two-sided Fisher’s exact test (SciPy 1.17.0). These exploratory comparisons were not tests of equivalence.

*P* < 0.05 was considered statistically significant. Statistical significance is indicated in the figures as follows: ns, not significant; *P < 0.05; **P < 0.01; ***P < 0.001; and ****P < 0.0001.

## 3 Results

### 3.1 Exploratory RNA sequencing and independent validation identify RPS3 upregulation in TKI-resistant CML

To screen candidate molecules associated with CML TKI resistance, transcriptome sequencing was performed in 11 patients (DIA, *n* = 3; GR, *n* = 3; R, *n* = 5). The resistance-focused R-versus-GR comparison used five R and three GR samples. Candidates selected using nominal *P* < 0.05 were intersected with RBPDB entries, yielding 28 exploratory RBP-associated candidates (**Figure 1A**, **Supplementary Table S1**). None met an adjusted *P*-value threshold of 0.05. Follow-up focused on the five candidates with higher expression in R than in GR: TRMT2A, DUS3L, TUT1, RPS3, and ZFR2.

**Figure 1:**
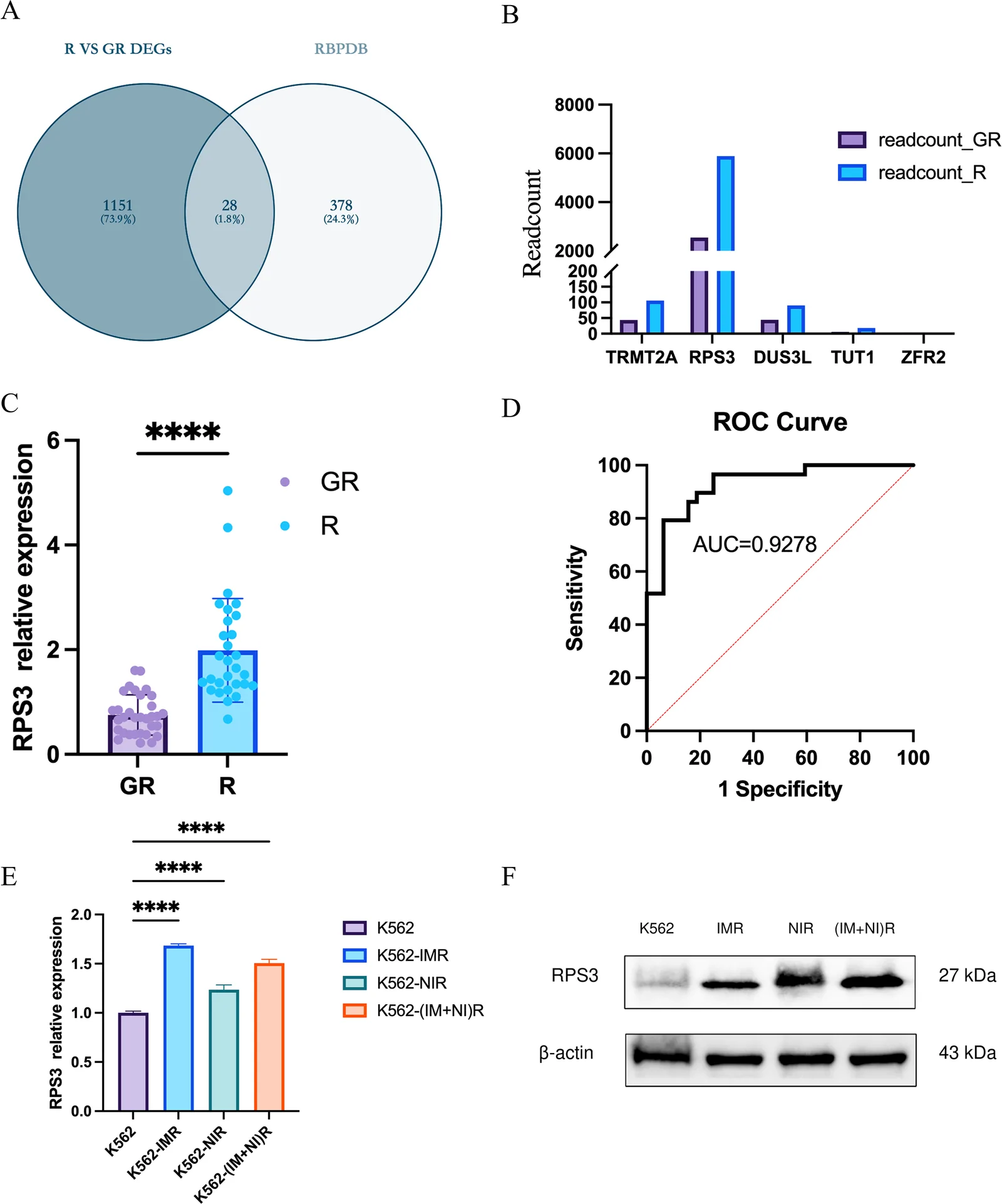
Exploratory RNA sequencing and independent validation identify RPS3 upregulation in TKI-resistant CML. **(A)** Venn diagram showing the intersection of the exploratory R-versus-GR candidate list (nominal *P* < 0.05) with RBPDB entries, yielding 28 RBP-associated candidates. RNA sequencing included 11 patients overall; this comparison used five R and three GR samples. **(B)** Normalized RNA-seq read-count abundance of the five candidates with higher expression in R than in GR. Expression abundance and published biological evidence informed the selection of RPS3 for follow-up. **(C)** RT-qPCR analysis of RPS3 mRNA expression in bone marrow/peripheral blood mononuclear cell samples from CML patients: good-response group (GR, *n* = 32) and resistant/poor-response group (R, *n* = 30). GAPDH was used as the internal control. **(D)** Receiver operating characteristic (ROC) analysis of RPS3 expression for distinguishing the resistant/poor-response group from good responders. The analysis used the same clinical expression cohort as **(C)** (R, *n* = 30; GR, *n* = 32), which was independent of the RNA-seq discovery cohort. **(E)** RT-qPCR analysis of RPS3 mRNA expression in parental K562 cells and resistant cell lines (K562-IMR, K562-NIR, and K562-(IM+NI)R). GAPDH was used as the internal control. **(F)** Western blot analysis of RPS3 protein expression in parental K562 cells and resistant cell lines. β-Actin was used as the loading control. Representative blots from three independent experiments are shown. For **(B)**, the RNA-seq groups comprised R, *n* = 5, and GR, *n* = 3. The patient numbers for **(C)** are specified above. Cell-based quantitative data are presented as mean ± SD from three independent experiments; **(F)** shows representative blots. ****P < 0.0001.

Among the five upregulated candidates, RPS3 had the highest normalized read-count abundance. Together with published evidence linking its extra-ribosomal functions to NF-κB signaling, cell survival, and drug resistance (22–24, 31–33), this motivated its selection for further investigation (**Figure 1B**). This prioritization did not imply that RPS3 was the only biologically relevant candidate. RT-qPCR in an independent clinical cohort of 30 resistant/poor-response patients and 32 good responders showed higher RPS3 mRNA expression in the R group (**Figure 1C**). All patients were in chronic phase; available clinical characteristics and TKI histories are summarized in **Supplementary File** CML_patient—Additional clinical information. Following confirmation of the expression difference, ROC analysis in the same cohort yielded an AUC of 0.9278 (95% CI: 0.8648–0.9908; *P* < 0.0001), indicating strong discrimination between the study-defined response groups (**Figure 1D**). RPS3 mRNA and protein expression were also increased in resistant cell models relative to parental K562 cells (**Figures 1E, F**).

### 3.2 RPS3 knockdown enhances the sensitivity of resistant cells to TKIs

To investigate the functional role of RPS3 in TKI resistance, stable RPS3-knockdown resistant cell lines were generated using lentivirus-mediated shRNA. RT-qPCR and western blotting showed that both RPS3 mRNA and protein levels were significantly decreased in knockdown cells, confirming successful establishment of the knockdown model (**Figures 2A, B**).

**Figure 2:**
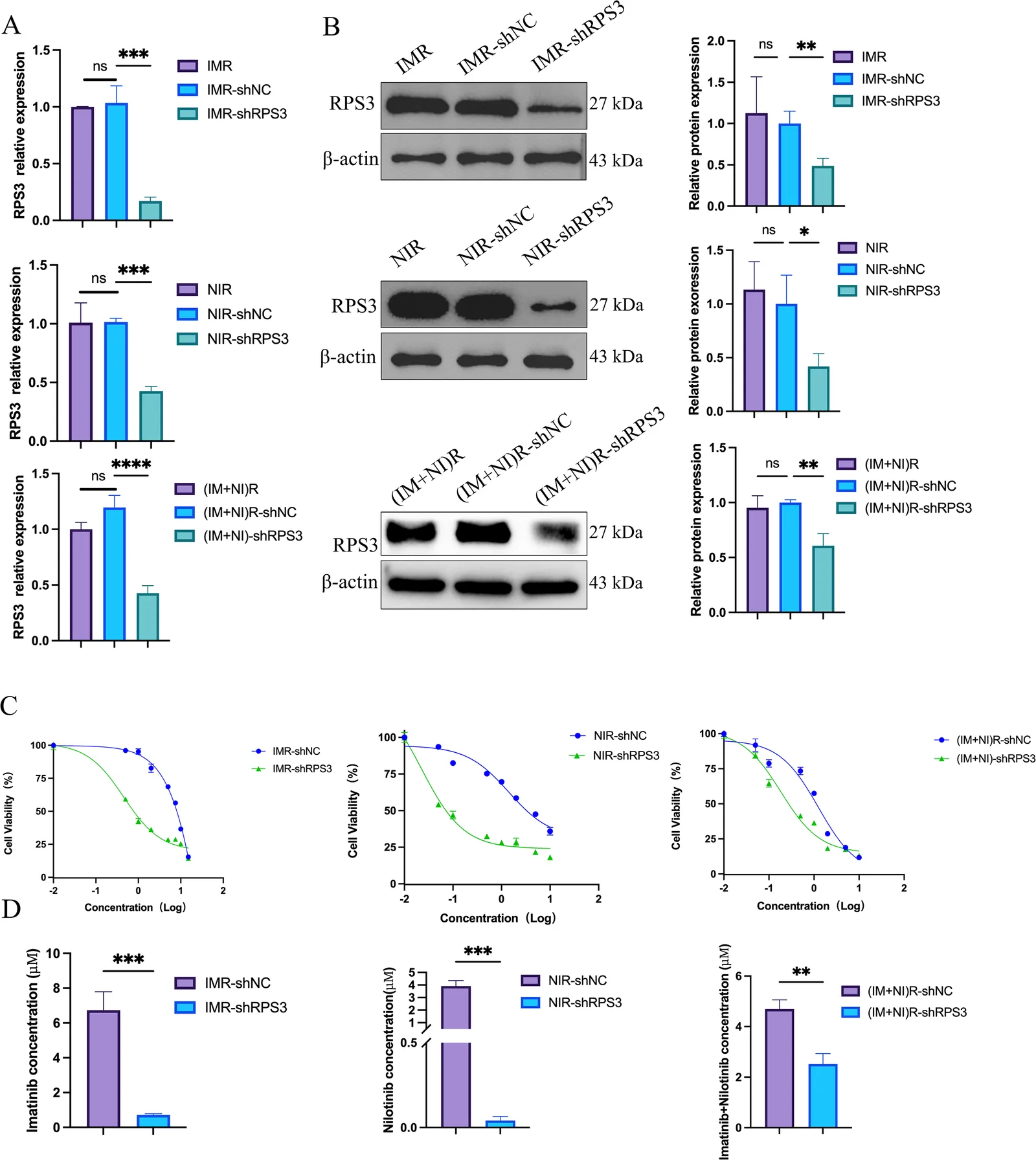
RPS3 knockdown enhances the sensitivity of resistant cells to TKIs. **(A)** RT-qPCR analysis of RPS3 mRNA expression in K562-IMR, K562-NIR, and K562-(IM+NI)R cells without transfection or transfected with shNC or shRPS3. GAPDH was used as the internal control. **(B)** Western blot analysis of RPS3 protein expression in resistant CML cells without transfection or transfected with shNC or shRPS3. β-Actin was used as the loading control. Representative blots and quantitative analysis from three independent experiments are shown. **(C)** CCK-8 dose–response curves of resistant CML cells transfected with shNC or shRPS3 after treatment with the corresponding TKI for 48 h. Curves were fitted using the normalized-response variable-slope nonlinear regression model described in the Methods. **(D)** Quantification of half-maximal inhibitory concentration (IC_50_) values calculated from the dose–response curves. Data are presented as mean ± SD (*n* = 3). *P < 0.05, **P < 0.01, ***P < 0.001, and ****P < 0.0001; ns, not significant. Multigroup comparisons with shNC were analyzed using ordinary one-way ANOVA followed by Dunnett’s multiple-comparisons test; comparisons limited to shNC and shRPS3 used an unpaired, two-tailed Student’s *t*-test. These group comparisons were applied to the IC_50_ estimates in **(D)**; **(C)** presents the fitted dose–response curves.

To determine the effect of RPS3 knockdown on TKI sensitivity in resistant cells, cell sensitivity to TKIs was assessed using the CCK-8 assay. After treatment with imatinib or nilotinib, RPS3-knockdown cells showed a greater reduction in viability, and the IC_50_ values were significantly lower than those of the corresponding shNC control groups. These results indicate that RPS3 knockdown enhances the sensitivity of resistant cells to TKIs (**Figures 2C, D**).

### 3.3 RPS3 knockdown enhances TKI-induced apoptosis and G_0_/G_1_-phase arrest

Because RPS3 knockdown significantly enhanced the sensitivity of resistant cells to TKIs, we further examined the effects of RPS3 knockdown on TKI-induced apoptosis and cell cycle distribution to explore the potential mechanisms by which RPS3 maintains the resistant phenotype.

Flow cytometric analysis with Annexin V/7-AAD double staining showed that after 48 h of treatment with imatinib or nilotinib, the proportion of Annexin V-positive cells was markedly increased in the RPS3-knockdown groups. These cells included Annexin V-positive/7-AAD-negative early apoptotic cells and Annexin V-positive/7-AAD-positive late apoptotic/dead cells. The total apoptosis rate was significantly higher than that in the corresponding shNC control groups. Notably, in the absence of TKI treatment, RPS3 knockdown did not cause a marked increase in spontaneous apoptosis, suggesting that RPS3 contributes to resistance to TKI-induced apoptosis, with limited effects on basal survival under the conditions tested (**Figures 3A–C**).

**Figure 3:**
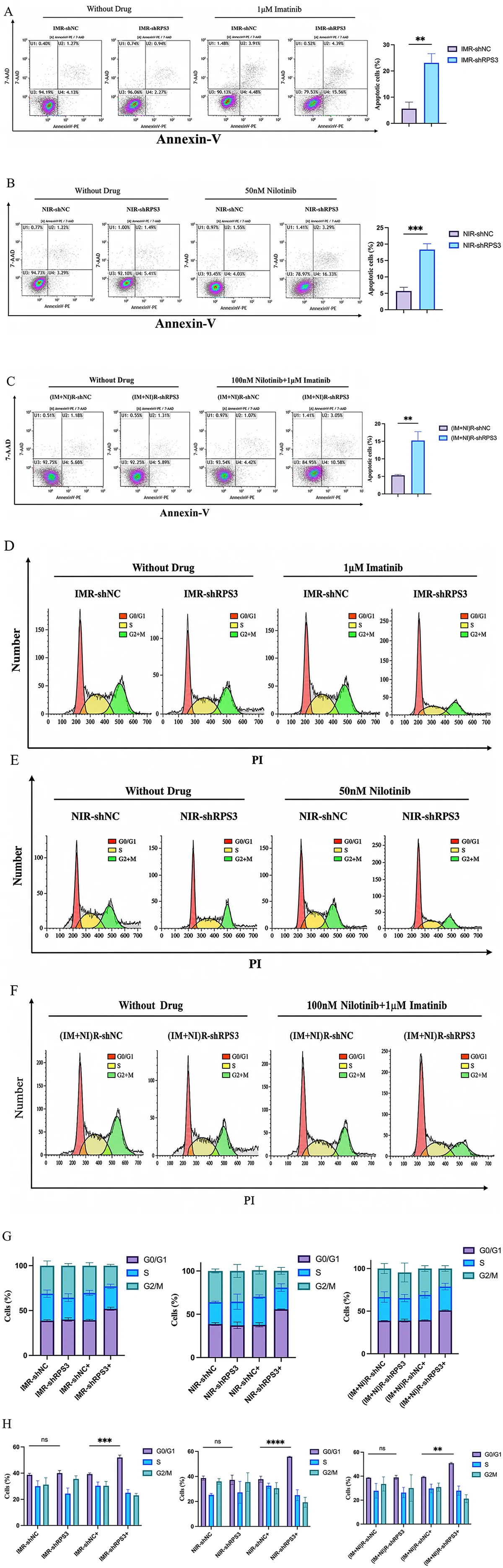
RPS3 knockdown enhances TKI-induced apoptosis and G_0_/G_1_-phase arrest. **(A–C)** Annexin V/7-AAD double staining and flow cytometry were used to evaluate the effect of RPS3 knockdown on TKI-induced apoptosis. **(A)** Representative flow cytometry plots of apoptosis in K562-IMR-shNC and K562-IMR-shRPS3 cells treated with or without 1 μM imatinib for 48 h. **(B)** Representative flow cytometry plots of apoptosis in K562-NIR-shNC and K562-NIR-shRPS3 cells treated with or without 50 nM nilotinib for 48 h. **(C)** Representative flow cytometry plots of apoptosis in K562-(IM+NI)R-shNC and K562-(IM+NI)R-shRPS3 cells treated with or without 100 nM nilotinib plus 1 μM imatinib for 48 h. Representative plots show both untreated and TKI-treated groups; the adjacent bar graphs quantify apoptosis in the TKI-treated shNC and shRPS3 groups. **(D–F)** PI staining and flow cytometry were used to evaluate the effect of RPS3 knockdown on TKI-induced changes in cell cycle distribution. **(D)** Representative cell cycle distribution plots of K562-IMR-shNC and K562-IMR-shRPS3 cells treated with or without 1 μM imatinib for 48 h. **(E)** Representative cell cycle distribution plots of K562-NIR-shNC and K562-NIR-shRPS3 cells treated with or without 50 nM nilotinib for 48 h. **(F)** Representative cell cycle distribution plots of K562-(IM+NI)R-shNC and K562-(IM+NI)R-shRPS3 cells treated with or without 100 nM nilotinib plus 1 μM imatinib for 48 h. **(G)** Stacked bar charts showing the proportions of cells in the G_0_/G_1_, S, and G_2_/M phases in each treatment group. **(H)** Quantitative analysis of the proportions of cells in the G_0_/G_1_, S, and G_2_/M phases. Within each panel, the cell models are K562-IMR, K562-NIR, and K562-(IM+NI)R from left to right. A plus sign after a group label denotes the corresponding TKI treatment specified in **(D–F)**. Data are presented as mean ± SD (*n* = 3). **P < 0.01, ***P < 0.001, and ****P < 0.0001; ns, not significant. Cell cycle phase distributions were analyzed separately for each cell model using ordinary two-way ANOVA with treatment group and cell cycle phase as the factors, including their interaction, followed by Tukey’s multiple-comparisons test comparing the four treatment groups within each phase. Each phase constituted a separate family of six pairwise comparisons.

Further cell cycle analysis showed that under TKI treatment, the proportion of cells in G_0_/G_1_ phase was significantly increased in RPS3-knockdown cells. In contrast, no obvious difference in cell cycle distribution was observed between groups in the absence of drug treatment. These results indicate that RPS3 downregulation enhances the response of resistant cells to TKI-induced cell cycle arrest (**Figures 3D–H**).

Together, these results indicate that RPS3 knockdown enhances TKI-induced apoptosis and G_0_/G_1_-phase arrest, with limited effects in the absence of TKI treatment under the conditions tested.

### 3.4 RPS3 knockdown reduces markers of NF-κB activation and MCL-1 and BCL-XL mRNA levels

The reported role of RPS3 in NF-κB transcriptional complexes (24), together with evidence linking NF-κB signaling to CML cell survival and TKI resistance (28–30), prompted us to examine whether RPS3 knockdown alters markers of NF-κB activation in resistant cells.

Western blotting showed that RPS3 knockdown markedly reduced phosphorylated p65 (p-p65) levels, whereas total p65 expression remained largely unchanged (**Figures 4A, B**). Immunofluorescence analysis further showed a significantly lower p65 nuclear/cytoplasmic ratio in RPS3-knockdown cells than in the corresponding shNC controls in both K562-IMR (*P* < 0.01) and K562-NIR (*P* < 0.001) cells (**Figures 4C, D**). These findings indicate reduced nuclear accumulation of p65 relative to its cytoplasmic distribution after RPS3 knockdown and, together with the reduced p65 phosphorylation, support attenuated NF-κB activation.

**Figure 4:**
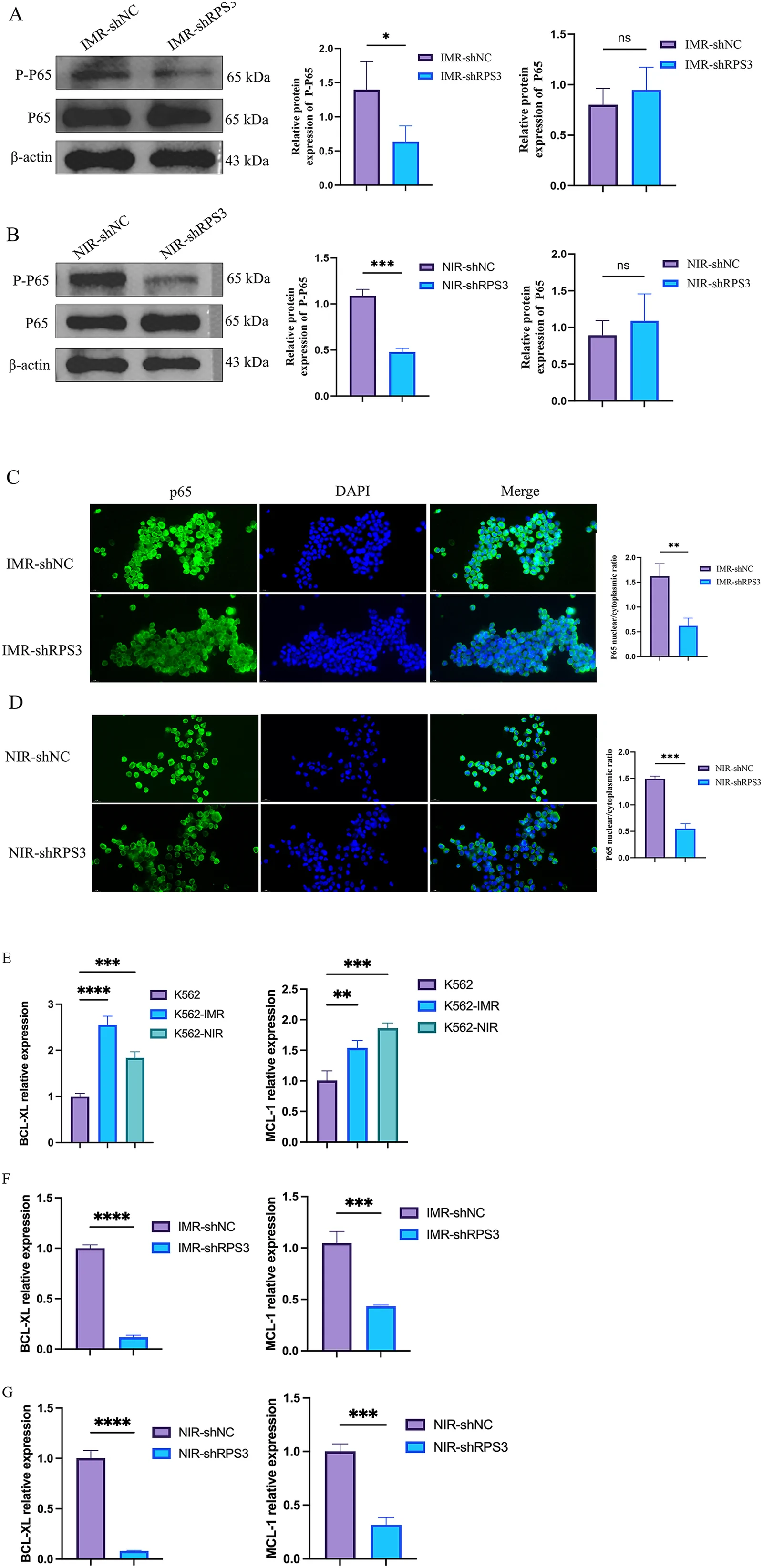
RPS3 knockdown reduces markers of NF-κB activation and MCL-1 and BCL-XL mRNA levels. **(A, B)** Western blot analysis of NF-κB signaling-related protein expression after RPS3 knockdown. **(A)** Phosphorylated p65 (p-p65) and total p65 protein levels, together with quantitative analysis, in K562-IMR-shNC and K562-IMR-shRPS3 cells. **(B)** p-p65 and total p65 protein levels, together with quantitative analysis, in K562-NIR-shNC and K562-NIR-shRPS3 cells. β-Actin was used as the loading control. **(C, D)** Representative immunofluorescence images and quantification of the p65 nuclear/cytoplasmic ratio in K562-IMR-shNC and K562-IMR-shRPS3 cells **(C)** and K562-NIR-shNC and K562-NIR-shRPS3 cells **(D)**. Green fluorescence indicates p65, and blue fluorescence indicates DAPI-stained nuclei. Integrated p65 fluorescence intensity (IntDen) was measured in the nuclear and whole-cell regions using ImageJ with a uniform threshold across the images being compared. The nuclear/cytoplasmic ratio was calculated for each cell as nuclear IntDen/(whole-cell IntDen − nuclear IntDen). Fifty cells per group were analyzed in each independent experiment, and their individual ratios were averaged to obtain one value per group for that experiment. Cell culture, treatment, staining, and imaging were performed separately in each of three independent biological experiments, with both shNC and shRPS3 groups included in every experiment. Data are presented as mean ± SD of the three experiment-level means (*n* = 3); individual cells were not treated as independent biological replicates. Comparisons between shNC and shRPS3 groups were performed using an unpaired two-tailed Student’s *t*-test. **P < 0.01 and ***P < 0.001 versus the corresponding shNC group. **(E)** RT-qPCR analysis of the mRNA expression levels of the anti-apoptotic genes BCL-XL and MCL-1 in parental K562 cells and resistant cell lines K562-IMR and K562-NIR. Each gene was analyzed separately using ordinary one-way ANOVA followed by Dunnett’s multiple-comparisons test, with parental K562 as the control. **(F)** RT-qPCR analysis of BCL-XL and MCL-1 mRNA expression in K562-IMR-shNC and K562-IMR-shRPS3 cells. For each gene, shNC and shRPS3 were compared using an unpaired, two-tailed Student’s *t*-test. **(G)** RT-qPCR analysis of BCL-XL and MCL-1 mRNA expression in K562-NIR-shNC and K562-NIR-shRPS3 cells. For each gene, shNC and shRPS3 were compared using an unpaired, two-tailed Student’s *t*-test. Data are presented as mean ± SD (*n* = 3). *P < 0.05, **P < 0.01, ***P < 0.001, and ****P < 0.0001; ns, not significant.

To explore a possible association with anti-apoptotic gene expression, we measured MCL-1 and BCL-XL mRNA levels. MCL-1 and BCL-XL mRNA levels were significantly increased in resistant cells, whereas RPS3 knockdown markedly reduced their expression (**Figures 4E–G**).

Together, these findings associate the increased TKI sensitivity of RPS3-knockdown cells with reduced markers of NF-κB activation and lower MCL-1 and BCL-XL mRNA levels, supporting an NF-κB-associated anti-apoptotic model of RPS3 function in resistant CML cells.

### 3.5 TNF-α counteracts the increased TKI sensitivity of RPS3-knockdown cells

Because RPS3 knockdown reduced markers of NF-κB activation and MCL-1 and BCL-XL mRNA levels, we used pharmacological perturbation to investigate the possible involvement of NF-κB signaling in TKI resistance. RPS3-knockdown cells were treated with TNF-α, which is used to stimulate NF-κB signaling, with or without BAY-11-7082. These agents were added simultaneously with the corresponding TKI.

CCK-8 dose–response analysis showed that TNF-α treatment increased the IC_50_ values of the corresponding TKIs in K562-IMR-shRPS3 and K562-NIR-shRPS3 cells. IC_50_ values were numerically higher with 20 ng/mL TNF-α than with 10 ng/mL TNF-α. Co-treatment with BAY-11–7082 reduced IC_50_ values toward those observed without TNF-α (**Figures 5A–F**). These findings indicate that TNF-α counteracts the increased TKI sensitivity of RPS3-knockdown cells and that BAY-11–7082 attenuates this effect.

**Figure 5:**
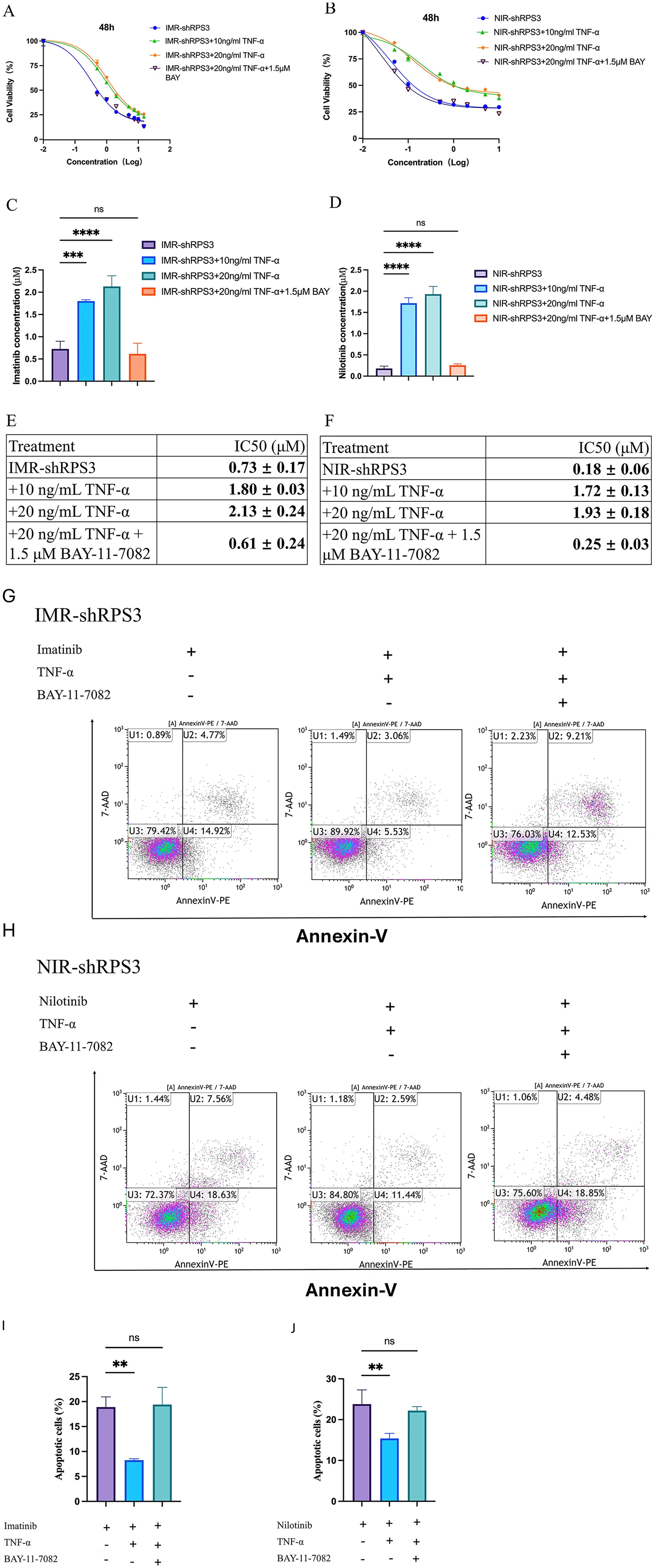
TNF-α counteracts the increased TKI sensitivity of RPS3-knockdown cells. **(A)** CCK-8 dose–response curves of K562-IMR-shRPS3 cells treated with imatinib alone or with 10 ng/mL TNF-α, 20 ng/mL TNF-α, or 20 ng/mL TNF-α plus 1.5 μM BAY-11–7082 for 48 h. Dose–response curves in **(A, B)** were fitted using the normalized-response variable-slope model described in the Methods. **(B)** CCK-8 dose–response curves of K562-NIR-shRPS3 cells treated with nilotinib under the same treatment conditions. **(C, D)** Quantitative analysis of IC_50_ values in K562-IMR-shRPS3 and K562-NIR-shRPS3 cells, respectively. Within each cell model, treatment groups were compared using ordinary one-way ANOVA followed by Dunnett’s multiple-comparisons test against the shRPS3+TKI group without TNF-α or BAY-11-7082. **(E, F)** Summary tables of IC_50_ values for each treatment group. These panels tabulate the IC_50_ results summarized in **(C)** and **(D)** and do not represent an additional statistical analysis. **(G, H)** Annexin V/7-AAD flow cytometry was used to evaluate apoptosis in K562-IMR-shRPS3 and K562-NIR-shRPS3 cells under the indicated treatment conditions. **(I, J)** Quantitative analysis of the percentages of apoptotic cells in K562-IMR-shRPS3 **(I)** and K562-NIR-shRPS3 **(J)**, corresponding to **(G, H)**, respectively. Data are presented as mean ± SD (*n* = 3). *P* < 0.05, **P < 0.01, ***P < 0.001, and ****P < 0.0001; ns, not significant..

Annexin V/7-AAD flow cytometry showed a corresponding pattern of apoptosis. TNF-α treatment reduced TKI-induced apoptosis in RPS3-knockdown cells, whereas co-treatment with BAY-11–7082 increased apoptosis toward the level observed in the shRPS3+TKI group (**Figures 5G–J**).

Together, the IC_50_ and apoptosis results provide pharmacological support for the involvement of NF-κB-associated signaling in the TKI response of RPS3-knockdown cells, complementing the changes in p65 phosphorylation and nuclear/cytoplasmic distribution.

### 3.6 BAY-11–7082 shows synergistic inhibitory effects with TKIs in resistant CML cells

We next examined whether BAY-11–7082 could enhance the inhibitory effects of TKIs in resistant cells. K562-IMR and K562-NIR cells were treated with concentration matrices of BAY-11–7082 and imatinib or nilotinib, respectively, and drug interactions were evaluated using the HSA model.

The imatinib/BAY-11–7082 combination yielded an HSA synergy score of 13.05 in K562-IMR cells, whereas the nilotinib/BAY-11–7082 combination yielded a score of 18.64 in K562-NIR cells. Both scores exceeded the predefined threshold for synergy. The heat maps also showed positive synergy scores within specific concentration regions (**Figures 6A, B**).

**Figure 6:**
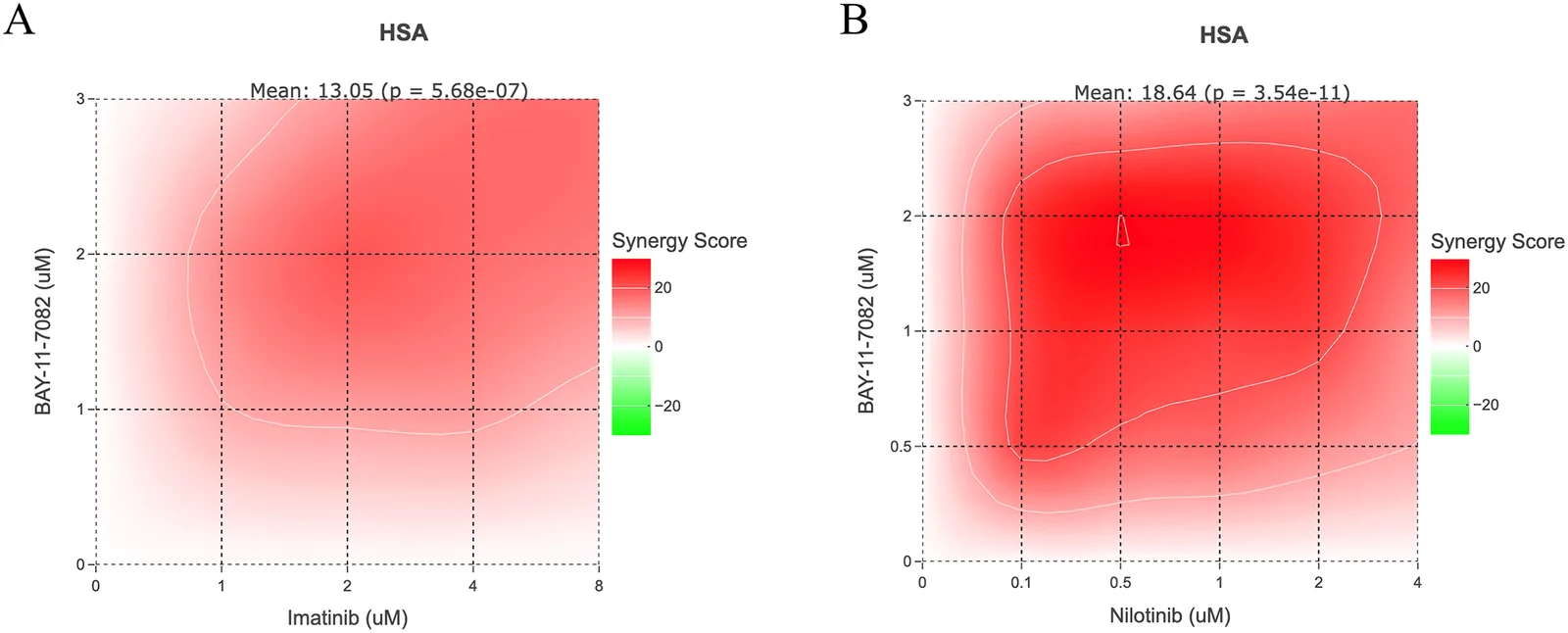
BAY-11–7082 shows synergistic inhibitory effects with TKIs in resistant CML cells. **(A)** HSA synergy heat map showing the combined effect of imatinib and BAY-11–7082 in K562-IMR cells. **(B)** HSA synergy heat map showing the combined effect of nilotinib and BAY-11–7082 in K562-NIR cells. According to commonly used SynergyFinder interpretation criteria, a synergy score >10 indicates synergy, a score between −10 and 10 indicates an additive effect, and a score <−10 indicates antagonism.

These findings identify BAY-11-7082/TKI combinations as candidates for further evaluation in resistant CML models and complement the evidence linking NF-κB-associated signaling to TKI sensitivity.

## 4 Discussion

This study identifies RPS3 as a contributor to TKI resistance in CML cell models by connecting clinical expression findings with functional evidence of altered drug sensitivity. RPS3 expression was elevated in samples from patients with resistance or poor responses and in resistant cell models, and RPS3 knockdown increased sensitivity to imatinib and nilotinib. The accompanying increases in TKI-induced apoptosis and G_0_/G_1_-phase arrest support a role for RPS3 in maintaining the resistant phenotype. Mechanistic analyses further linked these effects to reduced p65 phosphorylation, a lower p65 nuclear/cytoplasmic fluorescence ratio, and decreased MCL-1 and BCL-XL mRNA levels. Together, these findings place RPS3 within a proposed NF-κB-associated survival mechanism relevant to acquired TKI resistance.

RBPs provide a useful entry point for investigating resistance because they influence gene expression and cell fate, and several have been implicated in leukemia biology (15–21). The small RNA-seq cohort and the use of nominal *P*-values make the initial screen exploratory. In this study, RPS3 was selected from the exploratory transcriptome screen by considering its expression level, its upregulation in the resistant/poor-response group, and previous evidence linking it to cell survival and therapeutic resistance (22–24, 31–33). Other candidates were not systematically compared and may also be biologically relevant. RT-qPCR in a separate clinical cohort subsequently confirmed higher RPS3 mRNA expression in resistant/poor-response patients than in good responders. ROC analysis then quantified the ability of RPS3 expression to distinguish these groups within that cohort. These clinical findings, together with the effects of RPS3 knockdown on TKI sensitivity, connect the observed expression difference to a functional role in resistant CML cells. Additional cohorts are needed to assess generalizability, and prospective studies are required to evaluate prediction of future resistance.

All patients were in chronic phase, and classification incorporated their molecular-response history. Differences in treatment histories and unavailable baseline risk scores and reliable treatment timelines limit assessment of clinical confounding.

An informative feature of the functional results is their dependence on the treatment conditions. RPS3 knockdown had limited effects on apoptosis and cell cycle distribution in the absence of TKIs but enhanced both apoptosis and G_0_/G_1_-phase arrest after TKI exposure. This pattern suggests that RPS3 helps resistant cells withstand drug-induced stress under the conditions tested. The response to RPS3 depletion in both imatinib- and nilotinib-resistant models also supports its relevance across the two TKI settings examined. These findings motivate further evaluation of RPS3-directed sensitization strategies, while leaving effects on normal hematopoietic cells to be established.

RPS3 is a component of the 40S ribosomal subunit with reported extra-ribosomal functions (22, 23). Its identification as a non-Rel component of NF-κB complexes provides a basis for investigating its relationship with transcriptional survival programs (24). Previous studies have linked RPS3 to therapeutic resistance in adenoid cystic carcinoma and multiple myeloma and to XIAP regulation in breast cancer (31–33). In multiple myeloma, Chen et al. linked TRIP13/PKCδ-mediated RPS3 phosphorylation to canonical NF-κB activation and proteasome inhibitor resistance (33); whether this upstream mechanism operates in our CML models was not examined. Our findings extend this line of investigation to CML TKI resistance, connecting RPS3 depletion to both drug-response phenotypes and changes in p65 activation markers. This provides a disease- and treatment-specific context in which to investigate how the established molecular functions of RPS3 influence therapeutic response.

NF-κB signaling has already been implicated in CML cell survival and TKI resistance (25–30). Studies of PS1145, BAY-11-7082, and the IRAIN/NF-κB/CD44 pathway have also supported its therapeutic relevance (29, 30, 36). The present study adds RPS3 as a candidate regulator associated with this survival response. RPS3 knockdown reduced p65 phosphorylation and nuclear/cytoplasmic fluorescence ratios while increasing TKI sensitivity. These coordinated changes support a model in which RPS3 contributes to an NF-κB-associated anti-apoptotic state in resistant cells and provide a rationale for examining regulation at the level of an NF-κB-complex-associated protein.

Anti-apoptotic BCL-2 family members offer a plausible connection between altered survival signaling and TKI sensitivity (11, 37–39). MCL-1 inhibition has been reported to cooperate with imatinib in CML cells (37), and combined MCL-1 and BCR-ABL1 inhibition has shown synergistic effects in CML cell lines and patient-derived CD34^+^ stem/progenitor cells (38). BCL-XL has been implicated particularly in CML progression (39). In our models, MCL-1 and BCL-XL mRNA levels were elevated in resistant cells and decreased after RPS3 knockdown. Considered alongside the p65 findings and increased TKI-induced apoptosis, these results support including MCL-1 and BCL-XL as candidate downstream components of the proposed RPS3/NF-κB anti-apoptotic model.

The pharmacological experiments add a functional dimension to the signaling observations. TNF-α increased TKI tolerance and reduced TKI-induced apoptosis in RPS3-knockdown cells, whereas BAY-11–7082 counteracted these effects. The corresponding shifts in IC_50_ and apoptosis support the relevance of NF-κB-associated signaling to drug response after RPS3 depletion. Together with the biochemical and imaging results, these findings strengthen the proposed connection between RPS3, survival signaling, and resistance to TKI-induced apoptosis.

The proposed model also provides a framework for investigating how the multiple functions of RPS3 intersect with therapeutic resistance. In addition to its association with NF-κB, RPS3 has been reported to affect DNA repair, oxidative stress responses, and RNA-related processes (22–24). These functions may contribute alongside NF-κB-associated signaling. Future studies that integrate RPS3 interaction partners with transcriptional and translational responses could clarify which processes account for the drug-response phenotype and how they are coordinated. More broadly, recent work in AML identified MMRN1–EGFR-driven sialylglycan–Siglec signaling as a mechanism of leukemia stem-cell immune evasion (40). This provides a distinct example of survival mechanisms in myeloid malignancies, but it does not establish the same pathway in CML or connect it to RPS3; neither stem-cell persistence nor immune evasion was examined in the present study.

BAY-11–7082 showed synergistic inhibitory effects with imatinib and nilotinib in the corresponding resistant models under the HSA model. This finding complements the functional and signaling analyses by identifying a pharmacological combination with activity in the same resistant cell settings. Consistent with previous work on NF-κB-directed interventions in CML (29, 30, 36), it supports further investigation of approaches that combine TKI treatment with the disruption of survival signaling. In the context of the present study, the proposed RPS3/NF-κB/MCL-1/BCL-XL model offers a biological rationale for exploring such sensitization strategies in BCR::ABL1-independent resistance. RNA-directed delivery strategies are also being explored in hematologic malignancies. For example, a peptide–daunorubicin–siRNA nanoassembly targeting LILRB4 showed activity in preclinical AML models (41). This illustrates progress in targeted RNA delivery, while the feasibility, selectivity, and safety of delivering an RPS3-directed intervention in CML remain to be established.

Several limitations define the scope of these conclusions. Functional experiments were conducted primarily in resistant cell lines, and the findings require validation in primary patient cells and *in vivo*, including assessment of effects on normal hematopoiesis. NF-κB transcriptional activity was not directly measured. The direct regulatory relationships within the proposed RPS3/NF-κB/MCL-1/BCL-XL model remain unresolved: MCL-1 and BCL-XL were measured at the mRNA level, and protein measurements, transcriptional and promoter-occupancy assays, and genetic loss-of-function and rescue experiments are needed to establish their roles. The TNF-α/BAY-11–7082 experiments provide pharmacological support but do not establish NF-κB-specific causality; separating the ribosomal and extra-ribosomal contributions of RPS3 will also require further study. Finally, the combinations were evaluated *in vitro* using the HSA model. Additional interaction models and *in vivo* efficacy and tolerability studies are needed, and drug synergy alone does not establish dependence on the entire proposed axis. The therapeutic implications therefore remain preclinical.

In conclusion, this study connects elevated RPS3 expression in clinical samples with a functional contribution to TKI resistance in CML cell models. RPS3 knockdown enhances TKI-induced apoptosis and G_0_/G_1_-phase arrest, accompanied by reduced markers of NF-κB activation and lower MCL-1 and BCL-XL mRNA levels. The pharmacological findings further support the involvement of NF-κB-associated survival signaling. These results support a proposed RPS3/NF-κB/MCL-1/BCL-XL model and identify RPS3 as a candidate regulator for further investigation in BCR::ABL1-independent CML resistance, providing a rationale for strategies aimed at sensitizing resistant cells to TKIs.

## Data Availability

The RNA-seq data generated in this study are available from the corresponding author upon reasonable request.

## Supplementary material

The **Supplementary Material** includes the exploratory RBP-associated candidate screening results **Supplementary Table S1**, RT-qPCR primer and shRNA sequences **Supplementary Table S2**, reagent and antibody information **Supplementary Table S3**, and IC50 summaries **Supplementary Table S4**. Available clinical characteristics and recorded TKI histories for the validation cohort are provided in **Supplementary File** CML_patient—Additional clinical information, which supplements the originally submitted CML_patient file.
