# Supplementary material for: Role and mechanism of ribosomal protein S3 in tyrosine kinase inhibitor resistance in chronic myeloid leukemia
Source: Front Oncol. 2026 Oct 29;16:2005945. doi: 10.3389/fonc.2026.2005945 (PMC13754745; doi:10.3389/fonc.2026.2005945)
Supplement: Supplementary file 1 [file Table3.docx]

CML_patient Additional clinical information

This document supplements the originally submitted CML_patient file. Available clinical characteristics and recorded TKI histories of the clinical validation cohort. R denotes the study-defined resistant/poor-response group; GR denotes good responders. All 62 patients were in chronic phase (CP).

# A Cohort characteristics

| **Characteristic** | **R group** | **GR group** |
| --- | --- | --- |
| Patients, n | 30 | 32 |
| Chronic phase, n (%) | 30 (100) | 32 (100) |
| Age available, n | 30 | 32 |
| Age, median (range), years | 49 (21–81) | 51 (14–74) |
| Male, n / available (%) | 21 / 30 (70.0) | 18 / 32 (56.3) |
| Female, n / available (%) | 9 / 30 (30.0) | 14 / 32 (43.8) |
| Age and sex not reported, n | 0 | 0 |
| Latest TKI mentioned: Imatinib, n (%) | 10 (33.3) | 19 (59.4) |
| Latest TKI mentioned: Nilotinib, n (%) | 2 (6.7) | 4 (12.5) |
| Latest TKI mentioned: Flumatinib, n (%) | 7 (23.3) | 9 (28.1) |
| Latest TKI mentioned: Dasatinib, n (%) | 7 (23.3) | 0 (0.0) |
| Latest TKI mentioned: Olverembatinib, n (%) | 4 (13.3) | 0 (0.0) |
| No TKI switch, n (%) | 11 (36.7) | 32 (100) |
| History of TKI switching, n (%) | 19 (63.3) | 0 (0.0) |
| Sokal score | Not available | Not available |
| Reliable treatment duration | Not available | Not available |
| Complete milestone response classification | Not available | Not available |

Age and sex summaries include all patients. Exploratory comparisons: age, two-sided Mann–Whitney U test, P = 0.257; sex, two-sided Fisher’s exact test, P = 0.302. These results do not establish equivalence. Drug categories describe the latest recorded TKI; for patients with TKI switches, the treatment summary does not establish sampling relative to each switch. TKI switching describes treatment history and was not itself used as the criterion for group allocation.

# B Patient level recorded treatment history

CP, chronic phase; MMR, major molecular response. Treatment histories were reviewed in the medical records. “None (no TKI switch)” indicates continuous use of the listed TKI without switching to another TKI, rather than missing treatment-history information. All GR patients and 11 R patients had no TKI switch. For patients who switched TKIs, previous agents are listed in the documented sequence when available; for R16, previous first- and second-generation TKIs were recorded without specific agent names. Exact treatment durations are not presented because reliable dates were unavailable. Patient identifiers are anonymized.

## Resistant or poor response group

| **ID** | **Sex / age** | **Latest recorded TKI** | **Previous recorded TKI(s)** |
| --- | --- | --- | --- |
| R01 | Male / 60 | Olverembatinib | Imatinib → flumatinib |
| R02 | Female / 21 | Flumatinib | Nilotinib |
| R03 | Male / 65 | Flumatinib | None (no TKI switch) |
| R04 | Male / 21 | Flumatinib | Imatinib |
| R05 | Male / 26 | Dasatinib | Flumatinib |
| R06 | Female / 26 | Imatinib | None (no TKI switch) |
| R07 | Female / 28 | Nilotinib | Imatinib |
| R08 | Female / 67 | Imatinib | None (no TKI switch) |
| R09 | Male / 45 | Dasatinib | Imatinib |
| R10 | Male / 27 | Olverembatinib | Imatinib → flumatinib |
| R11 | Male / 52 | Imatinib | None (no TKI switch) |
| R12 | Female / 47 | Imatinib | Nilotinib |
| R13 | Female / 57 | Flumatinib | None (no TKI switch) |
| R14 | Male / 23 | Flumatinib | None (no TKI switch) |
| R15 | Male / 67 | Dasatinib | Imatinib |
| R16 | Male / 54 | Olverembatinib | First- and second-generation TKIs; agents unspecified |
| R17 | Male / 26 | Olverembatinib | Imatinib |
| R18 | Male / 33 | Imatinib | Imatinib → flumatinib → nilotinib |
| R19 | Male / 54 | Imatinib | None (no TKI switch) |
| R20 | Female / 31 | Nilotinib | None (no TKI switch) |
| R21 | Male / 81 | Imatinib | None (no TKI switch) |
| R22 | Male / 53 | Dasatinib | Flumatinib |
| R23 | Male / 41 | Dasatinib | Flumatinib |
| R24 | Female / 51 | Flumatinib | Imatinib |
| R25 | Male / 53 | Dasatinib | Flumatinib |
| R26 | Male / 37 | Flumatinib | None (no TKI switch) |
| R27 | Male / 35 | Imatinib | Nilotinib |
| R28 | Male / 65 | Dasatinib | Imatinib |
| R29 | Female / 71 | Imatinib | None (no TKI switch) |
| R30 | Male / 59 | Imatinib | Dasatinib |

## Good response group

| **ID** | **Sex / age** | **Latest recorded TKI** | **Previous recorded TKI(s)** |
| --- | --- | --- | --- |
| GR01 | Female / 65 | Nilotinib | None (no TKI switch) |
| GR02 | Male / 42 | Flumatinib | None (no TKI switch) |
| GR03 | Female / 50 | Flumatinib | None (no TKI switch) |
| GR04 | Female / 69 | Flumatinib | None (no TKI switch) |
| GR05 | Female / 28 | Imatinib | None (no TKI switch) |
| GR06 | Female / 37 | Imatinib | None (no TKI switch) |
| GR07 | Male / 38 | Imatinib | None (no TKI switch) |
| GR08 | Female / 49 | Imatinib | None (no TKI switch) |
| GR09 | Female / 52 | Imatinib | None (no TKI switch) |
| GR10 | Male / 65 | Flumatinib | None (no TKI switch) |
| GR11 | Male / 72 | Imatinib | None (no TKI switch) |
| GR12 | Male / 39 | Flumatinib | None (no TKI switch) |
| GR13 | Female / 16 | Flumatinib | None (no TKI switch) |
| GR14 | Male / 46 | Imatinib | None (no TKI switch) |
| GR15 | Male / 67 | Imatinib | None (no TKI switch) |
| GR16 | Female / 49 | Imatinib | None (no TKI switch) |
| GR17 | Female / 56 | Imatinib | None (no TKI switch) |
| GR18 | Male / 21 | Imatinib | None (no TKI switch) |
| GR19 | Male / 58 | Imatinib | None (no TKI switch) |
| GR20 | Male / 74 | Nilotinib | None (no TKI switch) |
| GR21 | Male / 49 | Nilotinib | None (no TKI switch) |
| GR22 | Male / 64 | Flumatinib | None (no TKI switch) |
| GR23 | Female / 64 | Imatinib | None (no TKI switch) |
| GR24 | Male / 65 | Imatinib | None (no TKI switch) |
| GR25 | Female / 61 | Imatinib | None (no TKI switch) |
| GR26 | Male / 37 | Imatinib | None (no TKI switch) |
| GR27 | Male / 30 | Imatinib | None (no TKI switch) |
| GR28 | Female / 14 | Imatinib | None (no TKI switch) |
| GR29 | Female / 62 | Flumatinib | None (no TKI switch) |
| GR30 | Male / 49 | Flumatinib | None (no TKI switch) |
| GR31 | Male / 63 | Imatinib | None (no TKI switch) |
| GR32 | Male / 68 | Nilotinib | None (no TKI switch) |

# C Recorded clinical context for the R group

| **ID** | **Clinical context recorded in the available notes** |
| --- | --- |
| R01 | Persistently high molecular burden after prior TKIs; response-related switch after MMR loss confirmed by clinical record review |
| R02 | Below MMR response level on nilotinib; subsequent improvement noted; response-related switch after MMR loss confirmed by clinical record review |
| R03 | No MMR during flumatinib treatment |
| R04 | Prior molecular increase (IS 0.2024%) prompted switch; response-related switch after MMR loss confirmed by clinical record review |
| R05 | Below MMR response level on flumatinib; response-related switch after MMR loss confirmed by clinical record review |
| R06 | Incomplete molecular response on imatinib |
| R07 | Below MMR response level during nilotinib treatment; response-related switch after MMR loss confirmed by clinical record review |
| R08 | No MMR on imatinib |
| R09 | Treatment switch recorded; mutation-negative result noted without assay details; response-related switch after MMR loss confirmed by clinical record review |
| R10 | Sequential TKI changes recorded; response-related switch after MMR loss confirmed by clinical record review |
| R11 | Prior MMR followed by molecular increase |
| R12 | Switch from nilotinib to imatinib recorded; response-related switch after MMR loss confirmed by clinical record review |
| R13 | No MMR during flumatinib treatment |
| R14 | No MMR recorded; milestone timing cannot be verified |
| R15 | Molecular increase prompted treatment switch; response-related switch after MMR loss confirmed by clinical record review |
| R16 | Recurrence after prior TKIs; response-related switch after MMR loss confirmed by clinical record review |
| R17 | Prior MMR loss prompted treatment switch; response-related switch after MMR loss confirmed by clinical record review |
| R18 | Inadequate response during sequential TKI treatment; response-related switch after MMR loss confirmed by clinical record review |
| R19 | No MMR and fluctuating molecular results |
| R20 | Prior MMR followed by molecular increase |
| R21 | MMR loss; financial barrier to switching |
| R22 | Treatment switch recorded; response-related switch after MMR loss confirmed by clinical record review |
| R23 | Inadequate response on flumatinib; response-related switch after MMR loss confirmed by clinical record review |
| R24 | Inadequate response on imatinib; response-related switch after MMR loss confirmed by clinical record review |
| R25 | MMR loss during flumatinib treatment; response-related switch after MMR loss confirmed by clinical record review |
| R26 | MMR loss; flumatinib continued |
| R27 | MMR loss prompted switch from nilotinib; response-related switch after MMR loss confirmed by clinical record review |
| R28 | MMR loss on imatinib; response-related switch after MMR loss confirmed by clinical record review |
| R29 | MMR loss during continued imatinib |
| R30 | Myelosuppression on dasatinib; inadequate response on imatinib |

Clinical record review confirmed that response-related TKI switches in the R group followed loss of MMR. R30 had a separately recorded intolerance-related switch because of myelosuppression on dasatinib, followed by inadequate response on imatinib. R21 remained on imatinib despite MMR loss because of a financial barrier to switching. R04, R17, and R23 had BCR::ABL1 IS ≤0.1% at sampling and were classified using their prior response history. The table combines recorded treatment summaries with the clarification of switch indications after clinical record review. Complete Sokal variables and reliable treatment timelines were unavailable.
